# OPTICAL+: a frequency-based deep learning scheme for recognizing brain wave signals

**DOI:** 10.7717/peerj-cs.375

**Published:** 2021-02-04

**Authors:** Shiu Kumar, Ronesh Sharma, Alok Sharma

**Affiliations:** 1 School of Electrical and Electronic Engineering, Fiji National University, Suva, Fiji; 2 STEMP, University of the South Pacific, Suva, Fiji; 3 Institute for Integrated and Intelligent Systems, Griffith University, Brisbane, Australia; 4 Laboratory for Medical Science Mathematics, RIKEN Center for Integrative Medical Sciences, Yokohama, Kanagawa, Japan

**Keywords:** Human-computer interaction (HCI), Brain wave, Long short-term memory (LSTM), Common spatial pattern (CSP), Motor imagery (MI), Informative frequency band (IFB)

## Abstract

A human–computer interaction (HCI) system can be used to detect different categories of the brain wave signals that can be beneficial for neurorehabilitation, seizure detection and sleep stage classification. Research on developing HCI systems using brain wave signals has progressed a lot over the years. However, real-time implementation, computational complexity and accuracy are still a concern. In this work, we address the problem of selecting the appropriate filtering frequency band while also achieving a good system performance by proposing a frequency-based approach using long short-term memory network (LSTM) for recognizing different brain wave signals. Adaptive filtering using genetic algorithm is incorporated for a hybrid system utilizing common spatial pattern and LSTM network. The proposed method (OPTICAL+) achieved an overall average classification error rate of 30.41% and a kappa coefficient value of 0.398, outperforming the state-of-the-art methods. The proposed OPTICAL+ predictor can be used to develop improved HCI systems that will aid in neurorehabilitation and may also be beneficial for sleep stage classification and seizure detection.

## Introduction

A human–computer interaction (HCI) system uses cutting-edge techniques for establishing direct communication between the human brain and a computer (Li et al., 2016). HCI has gained tremendous attention over the recent years with the major focus being in gaming and biomedical applications. An HCI system, also widely known as a brain–computer interface (BCI) system, converts the mental state (brain waves) of humans into computer commands which can be used by the disabled people to recover their environmental control capabilities (Wang et al., 2016). It can also be used to detect pre-seizure activities (Zhou et al., 2018) and sleep stages (Yulita et al., 2018). The human brain waves are usually captured using electroencephalography (EEG) sensors, with non-invasive sensors preferred over invasive sensors due to the fact that surgery is required for invasive sensors and those non-invasive sensors can be integrated into wearable devices (Mullen et al., 2013). However, the drawback of using non-invasive sensors is that it is prone to environmental noise, which results in the captured signal having a low signal-to-noise ratio (SNR). Therefore, it becomes quite a challenging task to recognize different categories of brain wave signals with high accuracy.

The automatic classification of EEG signals is a significant step towards making the use of EEG more practical in applications and less dependent on the experts. The electrical potentials generated by the brain for different specific tasks are recorded from the scalp using EEG sensors. These signals are well structured, which makes it appropriate for machine learning. Thus, a vast number of researchers have explored or proposed various traditional methods (Abibullaev & Zollanvari, 2019; Borhani et al., 2019; Chowdhury et al., 2019; Jiao et al., 2019; Kumar, Sharma & Tsunoda, 2019a, 2019b; Mumtaz et al., 2018; Utsumi et al., 2018; Wang et al., 2018; Xing et al., 2018; Xu et al., 2018; Zhang et al., 2018) aiming to build a system that has low complexity and a high recognition rate for classification of the brain wave signals. Brain wave signals have gained recognition for a number of applications such as neuro-rehabilitation (Chowdhury et al., 2019; Neurostyle, 2017; Zeng et al., 2017), sleep-stage classification (Radha et al., 2019; Xu et al., 2020), seizure detection (Gao et al., 2020; Zhou & Li, 2020), emotion recognition (Liu et al., 2020; Yang et al., 2019) and biometric recognition where identification of individuals is done using their brain wave signals. For example, in Damaševičius et al. (2018) the authors have proposed a biometric system that combines cryptography with EEG signals for recognizing different individuals. Several authors have also explored the possibilities of data compression (Rajasekar & Pushpalatha, 2020) for reducing the bandwidth required in data transfer and feature selection or reduction approaches such as wave atom transform (Martisius et al., 2013), feature selection using *F*-statistic values (Peng et al., 2020) and locally-robust feature selection approach (Yin et al., 2020).

Common spatial pattern (CSP) and its variants (Cheng, Lu & Wang, 2017; Ramoser, Muller-Gerking & Pfurtscheller, 2000) are the prevailing feature extraction methods for motor imagery (MI) EEG signal classification. Filter bank CSP (FBCSP) (Ang et al., 2008), discriminative FBCSP (DFBCSP) (Thomas et al., 2009), sparse filter band CSP (SFBCSP) (Zhang et al., 2015), and binary particle swarm optimization approach for frequency band selection (Wei & Wei, 2015) are few of the methods that utilize CSP for feature extraction. These methods have focused on using multiple filter bands and in some cases finding individual-dependent frequency bands that would produce good classification ability. Several other approaches have also been proposed using CSP for extracting potentially significant features from the EEG signals after performing certain analysis. However, the EEG features vary over time and differ significantly in different individuals (Blankertz et al., 2010). Thus, there has been an ever-increasing demand for robust and more general feature extraction techniques.

A number of approaches have been proposed based on deep learning (convolutional neural networks—CNN (Wu et al., 2019; Yuksel & Olmez, 2015) and long short-term memory network (LSTM) (Kumar, Sharma & Tsunoda, 2019a)), feature priority and selection (Li & Feng, 2019; Luo et al., 2016), empirical mode decomposition (Gaur et al., 2018; Mingai et al., 2016), wavelet packet decomposition (Yang et al., 2016), Hjorth parameters (Türk et al., 2017), tangent space mapping (Kumar, Mamun & Sharma, 2017) and channel selection (Ghaemi et al., 2017). While these approaches have aimed to increase the classification accuracy to some extent, their performance is still limited because the EEG signals acquired from the scalp using non-invasive sensors usually have low SNR. A signal with low SNR contains a lot of unwanted noise which degrades the performance of an algorithm in recognizing the different categories of the brain wave signals. One way to obtain an EEG signal with better quality, that is, a signal with good SNR is by using invasive sensors. However, invasive sensors require surgery, which is not preferred by many people and is rarely used for HCI applications. Therefore, there is a need to enhance the quality of the signal acquired using non-invasive sensors such that it contains useful information that can aid in recognizing the different categories of the MI EEG signals. Several methods have been proposed for artifact removal, amongst which is a recently proposed movement artifact removal approach for EEG analysis in sports exercise (Butkevičiūtė et al., 2019). However, one of the simple methods to remove the artifacts or unwanted noise is by filtering. The responsive frequency band differs from one individual to another (Ang et al., 2008; Kumar, Sharma & Tsunoda, 2017; Novi et al., 2007; Poli, Kennedy & Blackwell, 2007) due to different skull size, skin thickness and the fact that the way one individual thinks about a task differs from the way another individual thinks about the same task. Therefore, using a fixed filter band will not produce a signal of the best quality. Moreover, manually tuning the filter bank for each individual is a tedious and time-consuming task. Thus, a method that automatically finds the subject-dependent filter band that will produce the most responsive signal is desired. The authors in Wei & Wei (2015) have employed binary particle swarm optimization algorithm for selecting the best frequency band(s) from 10 overlapping sub-bands for the classification of motor imagery EEG signals. Although performance improvement has been achieved, multiple sub-bands can be selected which will increase the computation time of the system. On the other hand, the sub-bands are pre-defined and as such this approach might ignore some important information. In Wodecki, Michalak & Zimroz (2018), the authors have used progressive genetic algorithm (GA) for optimal filter design for vibration signal analysis. The informative frequency band (IFB) that contains the most information about the damage depends on many factors, such as kinematic structure, operational conditions, type of damage and its location within the machine and thus the progressive GA algorithm has been proposed for finding the IFB. The filter coefficients of a linear phase digital finite impulse response filter is optimized with no restriction on the search space of the filter coefficients, which will increase the time taken to search for the optimal filter. Therefore, in order to address these issues, we propose a simple yet an effective adaptive filtering approach to obtain the signal that is of the best quality and combine it with our previous work (Kumar, Sharma & Tsunoda, 2019a) to obtain a predictor called OPTICAL+ which is able to outperform the state-of-the-art methods. In OPTICAL+, we use GA to optimize the parameters of a Butterworth bandpass filter, which finds the most IFB for each individual using which the most responsive signal can be obtained. Filtering the signal using this frequency band aids in reducing the unwanted noise while retaining the useful information contained in the signal. Thus, a signal that captures most of the useful information needed to recognize the different categories of MI-EEG signals will be obtained. This helps in obtaining features that are discriminative. After filtering, the features generated using CSP and LSTM are used to train a support vector machine (SVM) classifier. This trained SVM model is then used to classify the test samples. The contributions of this work can be summarized as follows:

- A single adaptive Butterworth filter is employed that is tuned using the GA algorithm for finding the IFB specific to each individual, which results in an improved performance in terms of brain wave classification. The search space is kept to a minimum of three parameters (filter order and upper and lower cutoff frequencies) to be able to find the parameters of the adaptive filter in the shortest possible time.
- The adaptive filter is integrated with the OPTICAL predictor, resulting in a new predictor named OPTICAL+, which can enhance the overall classification ability of the system. The proposed adaptive filter can be easily integrated with other methods to offer a better classification performance.

The rest of the article is organized as follows. The next section describes the dataset used and the proposed method. The results are presented and discussed in “Results and Discussion”. Finally, the paper ends with conclusions and future recommendations.

## Materials and Methods

In this section, we present a brief description of the dataset that has been used to evaluate the proposed method, followed by a detailed description of the proposed OPTICAL+ predictor.

### Dataset description

The publicly available GigaDB dataset (Cho et al., 2017) has been used in this work. It contains EEG signals for MI tasks recorded from 52 healthy subjects (33 males and 19 females). Data was collected as previously described in Kumar, Sharma & Tsunoda (2019a). Specifically, data was acquired at a sampling rate of 512 Hz using 64 Ag/AgCl active electrodes placed around the scalp based on the international 10-10 system. The EEG signals were recorded for the following tasks: left and right hand MI tasks, eyeball movement left/right, eyeball movement up/down, eye blinking, jaw clenching, head movement, and rest state. For each subject, either 100 or 120 trials of each of the left and right hand MI tasks were recorded. Out of the 52 subjects, the data of 38 subjects are well-discriminated while the remaining subject’s data is less discriminated. In this work, we have used the EEG signals for the left and right hand MI tasks. A detailed description of the dataset can be found online (Cho et al., 2017).

### Methods

**Figure 1:**
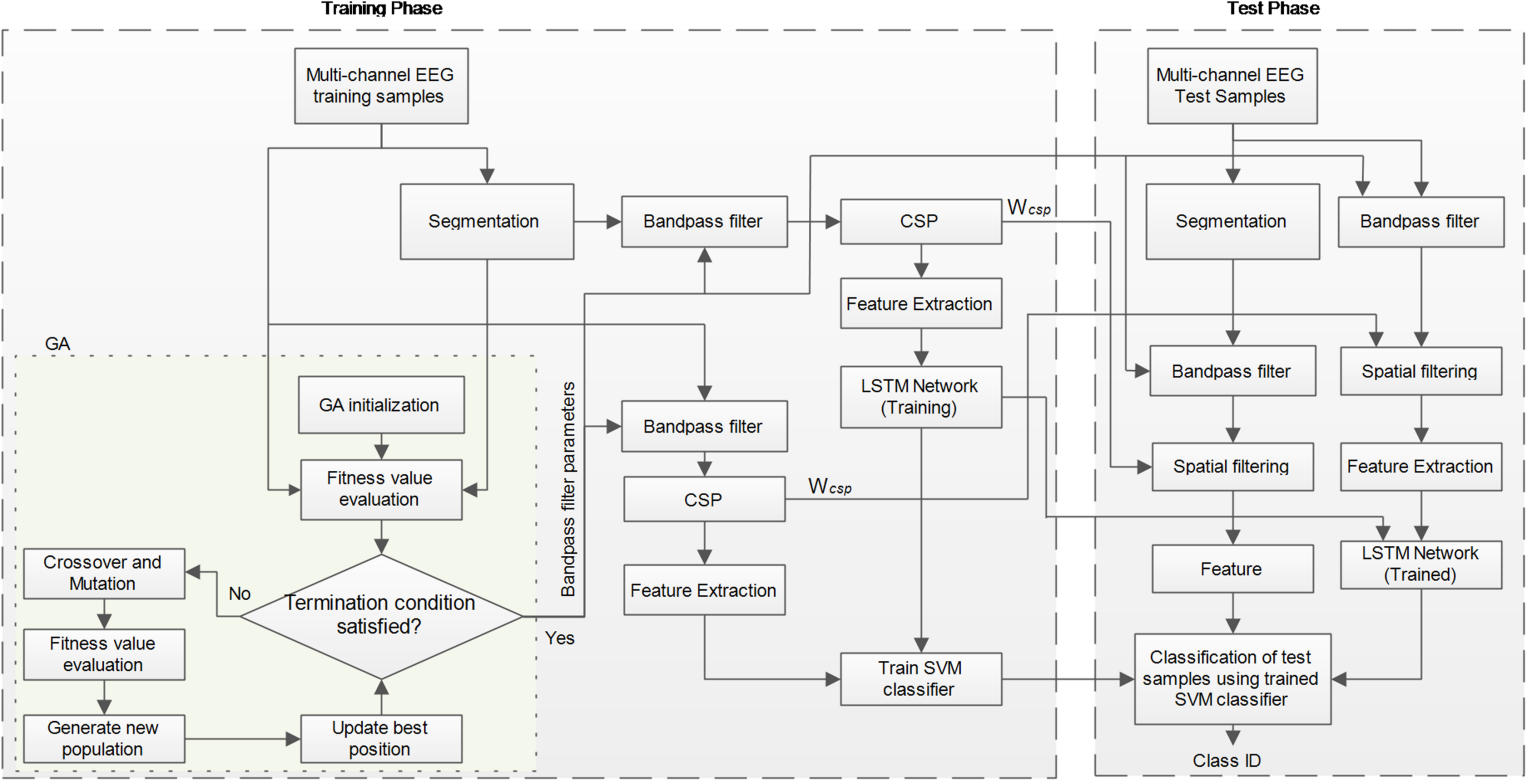
Conceptual framework of the proposed OPTICAL+ predictor.

Deep learning has been widely used in MI classification and has achieved satisfactory results. To make full use of the feature information, we propose a frequency-based approach embedded into the LSTM network, while also making use of the commonly used CSP approach for feature extraction. The overall framework of the proposed OPTICAL+ predictor is shown in Fig. 1. GA is first initialized with random parameters having a population size of 10. Two sets of spatial filters are then learned from the training samples. The first set of spatial filters is learned directly from the original training samples, while the second set of spatial filters is learned using the segmented data as shown is Fig. 1. CSP features are then obtained from both the spatially filtered data. The CSP features obtained from the first set of spatially filtered data becomes the first set of features to the SVM model. On the other hand, the CSP features extracted from the second set of spatially filtered data is fed as the sequence input to the LSTM network for training the model. The output of the LSTM network is a regression layer, which also becomes one of the features to the SVM model. Thus, the SVM model is trained using the training samples and evaluated using the evaluation samples. During this process of GA searching for the best filter parameters, 10-fold cross-validation is used for training and evaluating the performance of the selected filter parameters. Thus, the value of the fitness function is the average of the error rates obtained from the 10-folds. In this way, 10 fitness function values are obtained (one for each chromosome) during each phase of the fitness function evaluation. The GA search is carried out for 35 iterations unless an error rate of zero is achieved (in this case the search process of GA will stop) and the best parameters will be determined. Once the three filter parameters have been determined, all the training data is filtered using the adaptive filter that is learned, the spatial filters are again computed and the LSTM and SVM model is retrained. These spatial filters and trained models are then used during the test phase for classifying the new test samples. The following sub-sections present each of the phase of the proposed method in more detail.

### Adaptive filtering

Pre-processing the signals to obtain a clean signal is a fundamental concept for any signal processing approach. One of the key methods of obtaining a good quality EEG signal from the raw EEG data is through filtering. The EEG signal that will contain important MI task information usually lies between 0.5 Hz and 40 Hz. However, the actual frequency range that will provide important information about the different MI tasks varies amongst different individuals. This fact paves the way to develop methods that can automatically learn or find the IFB. While the use of multiple frequency bands has been proposed (Ang et al., 2008; Thomas et al., 2009), it increases the computational complexity of the system. Therefore, we propose the use of a single adaptive filter band using GA. The bandpass Butterworth filter has been utilized in this work due to the lower number of parameters that needs to be selected, which keeps the search space small. However, other filters can also be used with our proposed method. The three parameters that were tuned for the adaptive filter were the filter order, upper cutoff frequency and lower cutoff frequency. 10-fold cross-validation is performed on training data to search for the filter parameters that would result in the optimal performance of the proposed system. The adaptive filter parameters found are then used to filter the test data.

### Genetic algorithm

Genetic Algorithm (GA) is an iterative search procedure based on natural selection and genetics that is widely used for optimization and search problems. A *d* dimensional population of chromosomes representing the possible solutions is generated randomly, where *d* depends on the number of parameters to be optimized. In this work, we optimized *d* = 3 parameters (filter order, upper cutoff frequency and lower cutoff frequency). This first generation of the solutions is referred to as the parents. Only a few parents are then selected, from which children are generated using the cross-over method. Several methods such as Roulette wheel selection, rank selection, tournament selection, stochastic universal sampling and random selection can be used for selecting the parents. We have utilized the tournament selection approach for selecting the parents. Mutants are then formed by performing mutation on several randomly selected parents. The chromosomes that survive during the process of survivor selection become the parents in the next iteration. This process is repeated until the desired fitness condition or the desired number of iterations are reached. The chromosome that gives the best fitness value is selected and used to set the filter parameters of the adaptive filter for final training and testing.

### Common spatial pattern

Common Spatial Pattern (CSP) is one of the major approaches used for feature extraction of MI-EEG signals. It projects the signal onto a new time series, where the variance of one class of signals is maximized while the variance of the other class of signals is minimized. Refer to Kumar et al. (2016) for a detailed explanation of the CSP algorithm. Given an input signal ${E_n} \in {R^{C{\rm \; \times\; }T}}$, the spatially filtered signal can be obtained using (1), where *n* represents the *n*-th trial, *c* represents the number of channels and *t* is the number of sample points. The CSP variance-based features are then extracted using (2), where $\hat E_n^i$ represents the *i*-th row of ${\hat E_n}$. In this work, we have selected 6 spatial filters (*m* = 3), therefore, 6 CSP variance-based features are obtained.

(1) $${\hat E_n} = W_{CSP}^T{\rm \; \; }{E_n}$$

(2) $${F_n} = \log \left( {\displaystyle{{{\mathop{\rm var}} (\hat E_n^i)} \over {\mathop \sum \nolimits_{j = 1}^{2m} {\mathop{\rm var}} (\hat E_n^j)}}} \right)$$

### Long short-term memory network

**Figure 2:**
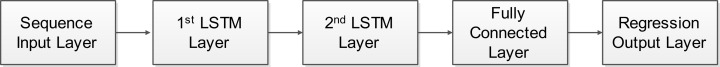
The LSTM network architecture used in OPTICAL+ predictor.

**Figure 3:**
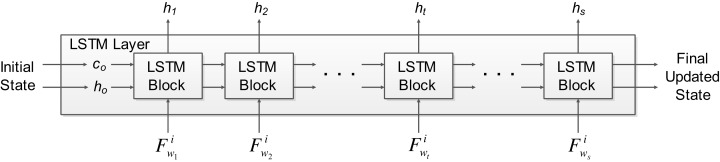
The LSTM layer architecture used in OPTICAL+ predictor.

Deep learning has been increasingly applied for solving real-life applications with good performance. Usually, deep learning methods such as convolutional neural networks were applied to image data. However, recurrent neural networks (RNN) such as LSTM are used for learning time series data. The long-term information of time series data can be stored by the LSTM network, and later used together with the current data to generate the output. The EEG signal architecture comprises of common temporal patterns when MI tasks are not performed. Therefore, non-temporal techniques may not be able to achieve optimal performance due to the shortfall that they will not be able to exploit the dependency between the time steps (i.e., current and past data) (Radha et al., 2019). Recurrent models such as LSTM are able to tackle this problem that normal RNN’s can’t by learning short-term dependencies for a very long time. Thus, we utilize an LSTM network in this work, which is a RNN that has an LSTM layer. The LSTM network architecture used in this work is shown in Fig. 2. The input to the network is a sequence input, followed by 2 LSTM layers, the fully connected layer and a regression layer at the output. The sequence input layer is used to provide the time series or sequence input to the network. The LSTM layer learns long-term dependencies between a series of sequence data. The LSTM layer architecture used in this work is shown in Fig. 3, which shows how the *d*-dimensional sequence input matrix *F* with a length of *s* flows through the LSTM layer. The $F_{{w_j}}^i$ represents the sequence input where *i* denotes the *i*-th feature obtained from the *j*-th windowed segment of the respective trial (details on how to obtain sequence input matrix can be found in our previous work (Kumar, Sharma & Tsunoda, 2019a)). The *h* and *c* denote the hidden/output states and cell states, respectively. The initial state of the network and the first sequence input $F_{{w_1}}^i$ becomes the input for the first LSTM block, which computes the first output *h*_1_ and *c*_1_ (updated cell states). The LSTM blocks in between the first and last LSTM block takes $F_{{w_1}}^i$ sequence input and current states of the network (*c*_t*−*1_, *h*_t−1_) for the *t*-th LSTM block for computing the updated output state *h*_t_ and the updated cell state *c*_t_. Information that is learned from previous sequence inputs are contained by the cell states. Information is added or removed (update controlled using gates) from the cell states at each step of the sequence input.

**Figure 4:**
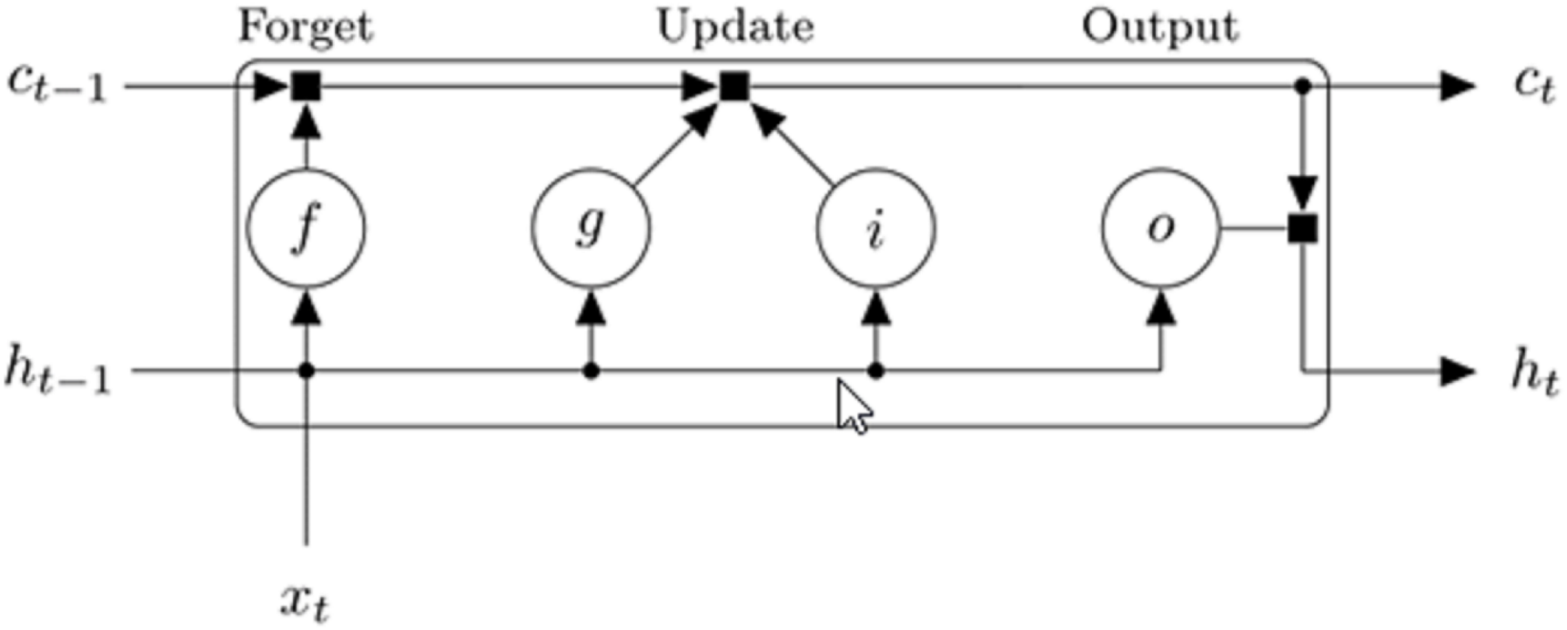
Diagram showing the flow of data through the LSTM block for *t*-th step of the input sequence (MathWorks, 2018).

An LSTM architecture usually consists of four main parts; the input gate, memory cell, forget gate and the output gate. The values (states) are remembered or stored by the memory cells for either short or long times. The function of the input gate is to control the degree to which new data or information can flow into the cell of the LSTM layer, the purpose of the forget gate is to control the degree to which certain value or information remains in the cell of the LSTM layer, while the function of the output gate is to control the amount of stored information that is utilized for computing the output activation. Figure 4 shows how the data flows through the LSTM block for the *t*-th step of the input sequence. The input weights ***W***, recurrent weights ***R*** and bias ***b*** are the weights that are learned by the LSTM layer.

The cell state at step *t* of the input sequence is calculated by ${C_t} = {f_t} \cdot {c_{t - 1}} + {i_t} \cdot {g_t}$ while the hidden/output state is calculated by ${h_t} = {o_t} \cdot {\sigma _c}\left( {{c_t}} \right)$ ${{\rm h}_{\rm t}} = {{\rm o}_{\rm t}}\cdot {{\rm \sigma }_{\rm c}}\left( {{{\rm c}_{\rm t}}} \right)$, where *i*, *f*, *g* and *o* denotes the input gate, forget gate, cell candidate and output gate, respectively. The (·) operator represents the element-wise multiplication (Hadamard product operator) and σ_*c*_ denotes the state activation function. Each of the components at sequence input *t* is calculated using Eqs. (3)–(6). The tangent hyperbolic function has been used as the state activation function, ${{\rm \sigma }_{\rm c}}\left( {\rm x} \right) = {\rm tanh}\left( {\rm x} \right)$ while the sigmoid function is used as the gate activation function, ${{\rm \sigma }_{\rm g}}\left( {\rm x} \right) = {\left( {1 + {{\rm e}^{ - {\rm x}}}} \right)^{ - 1}}$.

(3) $${i_t} = {\sigma _g}\left( {{W_i}{x_t} + {R_i}{h_{t - 1}} + {b_i}} \right)$$

(4) $${f_t} = {\sigma _g}\left( {{W_f}{x_t} + {R_f}{h_{t - 1}} + {b_f}} \right)$$

(5) $${g_t} = {\sigma _c}\left( {{W_g}{x_t} + {R_g}{h_{t - 1}} + {b_g}} \right)\;$$

(6) $${o_t} = {\sigma _g}\left( {{W_o}{x_t} + {R_o}{h_{t - 1}} + {b_o}} \right)$$

## Results and Discussion

In order to evaluate the feasibility and performance of the proposed system, experiments have been conducted using the GigaDB dataset. These experiments are conducted using Matlab running on a personal computer at 3.3 GHz with Intel(R) Core(TM) i7 processor.

The GigaDB dataset is used to evaluate the proposed method as it has a large number of individuals (52 individuals) and would generalize well for comparing the performance of the different methods. We extracted 2 s window signal after the visual cue was presented to obtain trial data for the different MI tasks (left hand and right hand) as done in other related works (Cho et al., 2017; Kumar & Sharma, 2018; Kumar, Sharma & Tsunoda, 2017, 2019b; Zhang et al., 2017). Basic pre-processing is performed, where common average referencing is applied to all the trials.

The performance measures that are used to evaluate the performance of the proposed system are the error rate (% of samples that are misclassified), sensitivity, specificity, and Cohen’s kappa index (κ). The error rate shows the percentage of trials that are incorrectly classified. The sensitivity and specificity shows the ability of the classifier or model to correctly classify the positive and negative trials, respectively. The Cohen’s kappa index is used to measure the reliability of the classifier and a higher value signifies that the system is more reliable.

## Results

For a fair comparison, all the methods have been implemented and the results obtained using 10 × 10-fold cross-validation scheme is reported. Table 1 shows the comparison of the 10 × 10-fold cross-validation results of the proposed OPTICAL+ predictor with the other state-of-the-art methods. It can be noted from Table 1 that the proposed method outperformed all the other methods in terms of the error rate, sensitivity, specificity and κ. It shows that our proposed method is reliable and more robust as it achieved the highest κ value. Our proposed method outperformed the top performing OPTICAL method by 1.40%, 1.30%, 1.60%, and 6.42% in terms of the error rate, sensitivity, specificity and κ, respectively. The results for the individual subjects can be found in the Supplemental Materials. Out of the 52 subjects, 21 subjects achieved the lowest error rate using the proposed approach. This is the highest number of subjects achieving the lowest error rates using a particular method with I-DFBCSP achieving the second-highest number of subjects (10 subjects) with the lowest error rates. Compared to the OPTICAL method, the error rates for 32 subjects improved using the OPTICAL+ method with the highest improvement being a 10% decrease in the error rate (subject 48).

**Table 1:** 10 × 10-fold cross-validation results of the different methods.

Method	Number of filters	Error rate	Sensitivity	Specificity	κ
CSP	1	34.07	0.664	0.651	0.343
DFBCSP [1]	4	38.46	0.619	0.616	0.266
SBLFB [3]	17	33.88	0.666	0.658	0.339
CSP-TSM [6]	1	35.02	0.654	0.647	0.323
SFTOFSRC [8]	16	36.80	0.623	0.531	0.291
SS-MEMDBF [11]	1	37.09	0.633	0.624	0.297
TFPO-CSP [26]	1	32.76	0.684	0.674	0.383
I-DFBCSP [28]	4	33.31	0.669	0.663	0.364
OPTICAL [13]	1	31.81	0.688	0.675	0.374
OPTICAL+	1	30.41	0.701	0.691	0.398

**Figure 5:**
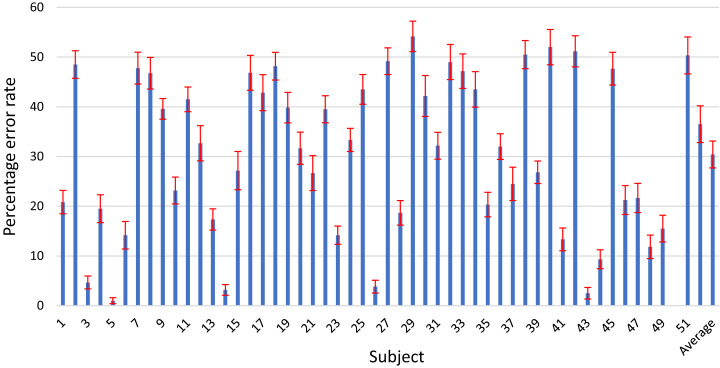
Error rates of the individual subjects for the proposed OPTICAL+ predictor.

We also present the results of the individual subjects for our proposed OPTICAL+ predictor in Fig. 5, where the error bars represent the 95% confidence interval. The results of the individual subjects for the other competing methods can be found in the Supplemental Material. It can be noted that our proposed method achieved the lowest width of the 95% confidence interval for 15 out of 52 subjects. This number is higher than that of any other competing methods. Over 96% of the subjects have a width of 95% confidence interval of less than ±3.75%. This shows that our proposed method can produce similar results if repeated under the same conditions. Thus, we can say that the proposed OPTICAL+ predictor is reliable as it achieved the lowest average width of 95% confidence interval showing that the proposed OPTICAL+ predictor has the lowest variation around its reported statistical mean.

## Discussion

The proposed OPTICAL+ predictor outperformed all the other state-of-the-art methods. The GigaDB dataset has been used to evaluate all the methods as it has a large number of subjects (52 subjects) and using this dataset shows the reliability and robustness of the approaches. Reliability in this work specifically refers to the degree to which a test produces similar results under consistent conditions and robustness refers to the strength of the approach. It is worth noting that approaches such as DFBCSP, SFTOFSRC, CSP-TSM and SS-MEMDBF have shown to perform well on small datasets (BCI Competition datasets). However, they did not perform well on the GigaDB dataset. On the other hand, the proposed OPTICAL+ predictor generalized well compared to these methods, showing that it is a more reliable and robust predictor for predicting the MI-EEG signals since it achieved the highest κ value. We say that our proposed system is more reliable because it is able to generate similar results under consistent conditions obtaining the lowest average standard deviation of 9.06 in comparison with the other competing methods. On the other hand, we argue that our approach is more robust in comparison to other methods as it is able to perform well (obtaining lowest error rate) for a substantial number of subjects (21 out of 52 subjects). This is considerably higher than the other methods as the number of subjects for which the competing methods obtained the lowest error rate are as follows: CSP—2, SBLFB—4, TFPO-CSP—7, I-DFBCSP—10 and OPTICAL—7.

Moreover, to show that the performance improvement achieved by our proposed OPTICAL+ predictor is significant, paired t-test with a significance level of 0.01 has been performed. The results of the individual subjects for the proposed OPTICAL+ predictor and the results of the individual subjects for the OPTICAL and TFPO-CSP predictors have been used to perform the paired *t*-test. The *p*-values obtained were 0.0015 and 0.0009, respectively. This indicates that a significant performance improvement has been achieved using the proposed OPTICAL+ predictor as the *p*-values obtained are <0.01.

**Figure 6:**
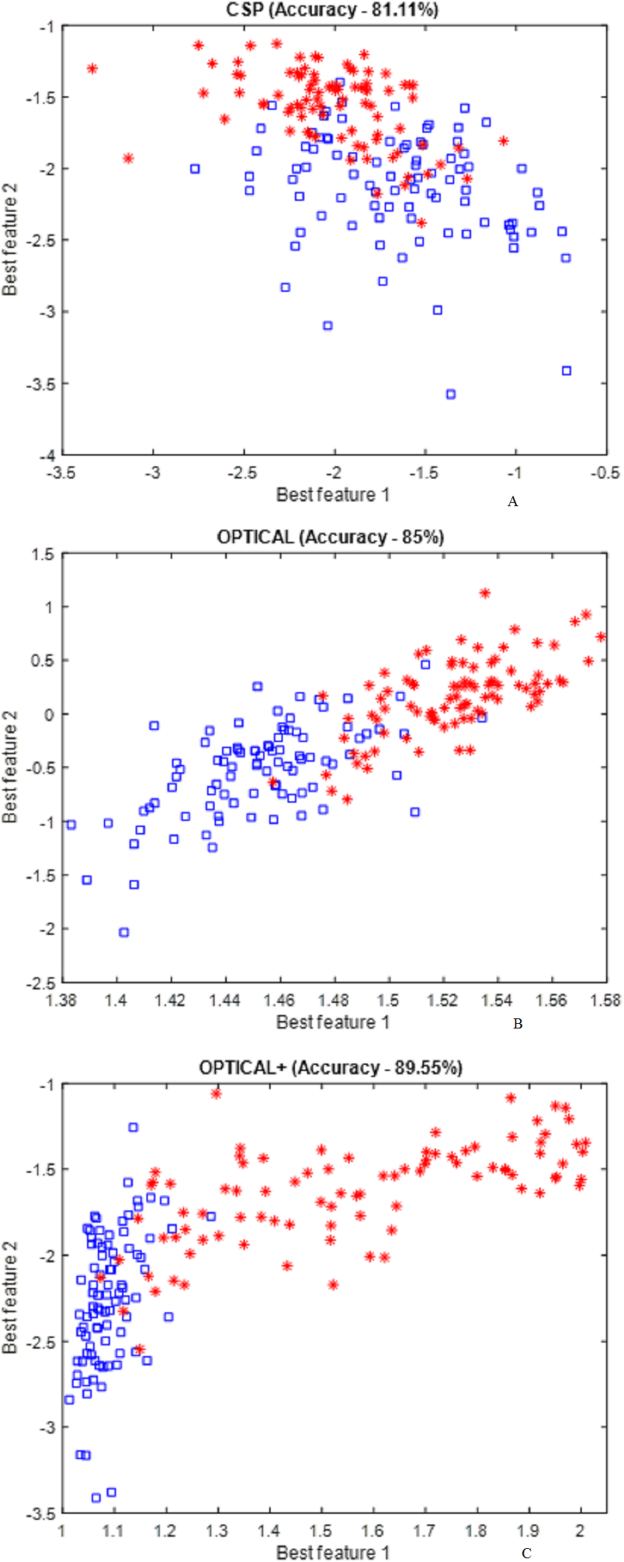
Distribution of the best two CSP features obtained using conventional CSP approach and proposed method.

**Figure 7:**
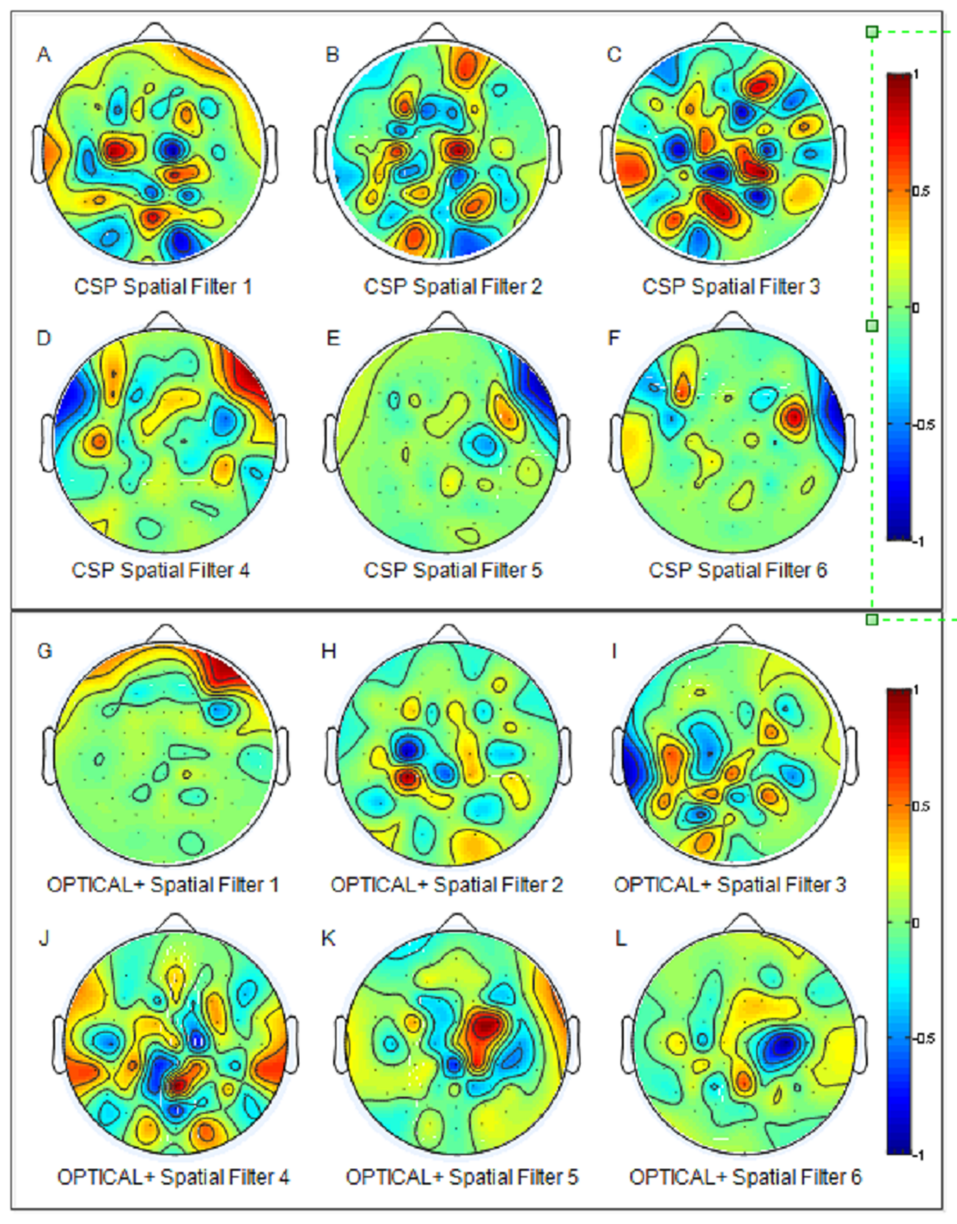
The six spatial filters learned by the conventional CSP approach (A–F) and the proposed OPTICAL+ predictor (G–L) for subject 48 (for one of the trial runs).

Figure 6 presents the distribution of the best two features for the CSP , OPTICAL and the proposed OPTICAL+ method that was obtained using one of the trial runs of subject 48. It can noted from Fig. 6 that more separable features are obtained by the proposed OPTICAL+ predictor in comparison to the conventional CSP and OPTICAL methods, thus, accounting for the enhanced performance of the proposed OPTICAL+ predictor. This is due to the optimization of the filter parameters, which results in the signal containing significant information while filtering out most of the redundant information. This leads to learning better CSP spatial filters that project data onto a new time series which are more separable, thus resulting in improved performance. The 6 spatial filters learned from one of the trial runs of subject 48 are shown in Fig. 7 for with and without optimization of the filter parameters. Figure 7 demonstrates that the CSP spatial filters learned when the filter parameters are optimized (OPTICAL+ spatial filters) were better than the CSP spatial filters learned when the filter parameters were not optimized as they were able to project the data to a new time series with the projected signal having more discrimination between the two MI tasks. This is evident, as Fig. 6 shows that the features learned from OPTICAL+ are more separable, resulting in higher classification accuracy.

**Figure 8:**
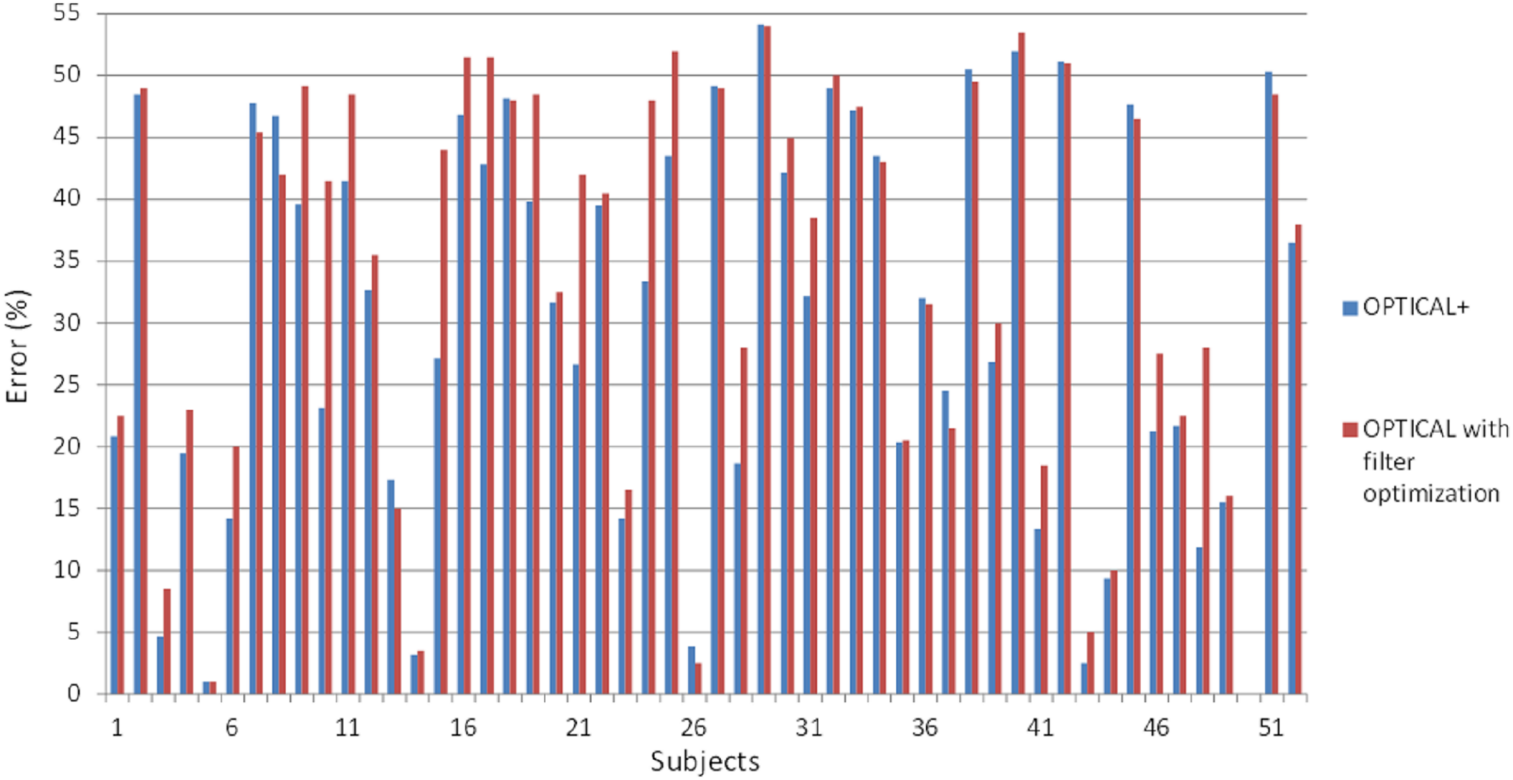
The error rates of OPTICAL predictor with optimization of the filter parameters and OPTICAL+ predictor.

Furthermore, we also performed experiments whereby we optimized the filter parameters followed by the optimization of the LSTM network using Bayesian optimization. The results for the individual subjects compared with the proposed OPTICAL+ predictor is shown in Fig. 8. The error rate for most of the subjects increased compared to the OPTICAL+ predictor. The overall performance of the system did not improve as the average error rate increased to 33.75%. This may be mainly due to over-fitting as both the filter parameters and the LSTM networks hyper-parameters were optimized consecutively and as such, this approach was not adopted. In future, we will perform further experiments to see if performance improvements can be achieved by simultaneously optimizing both the filter parameters and the LSTM hyperparameters.

Further works will be carried out in future where multiple sub-bands will be tested. Also, since convolutional neural network (CNN) has gained a lot of attention over the recent years, we will evaluate the use of CNN for MI-EEG signal classification by developing a hybrid model utilizing CNN with OPTICAL+. Moreover, CNN works well on image data, therefore, method such as DeepInsight (Sharma et al., 2019) can be used to transform the EEG signal to image before being fed as input to the CNN model. To add on, we have employed an LSTM network with peephole connections in this work. However, in future we will also consider the LSTM network with gated recurrent units, multiplicative LSTM (Krause et al., 2017) and LSTM networks with attention. All models trained in this work are subject-dependent, which is in agreement with the related works. Moreover, we have used GA in this work to demonstrate the significance of optimizing the filter band parameters. However, any other optimization algorithm can be employed with the proposed OPTICAL+ predictor. Furthermore, in future, we also intend to test how the proposed method would perform on other EEG signal classification tasks, such as sleep stage classification and seizure classification.

Moreover, although our proposed approach has outperformed other competing methods on various performance measures, it does have some limitations. One of the limitations of the proposed OPTICAL+ predictor is that we have used a population size of 10 with three parameters to be optimized. This results in an average time of about 5 minutes for finding the optimal parameters of the adaptive filter. This means that if any other filter that will require more parameters to be tuned is used, then it will lead to even higher training time. Thus, higher computational resources will be required for training. However, we believe this is not a major limitation due to the fact that training can be done offline and the trained model can then be deployed to the preferred device to be used for real-time operation. This is because the time required to process and classify a test signal using the trained model is 6.96 ms. There is also a possibility that during the process of searching for the optimal filter parameters, the search might get stuck with the local maximum and is not able to find the optimal solution. To alleviate this possibility, higher population size can be used. However, this will again result in higher computational time and a need for higher computational resources.

## Conclusions

The proposed method has been able to successfully optimize the filter band parameters that accounts for its improved performance in comparison to the other state-of-the-art methods. OPTICAL+ achieved the lowest error rate of 30.41%, which is an improvement of 1.40% compared to the OPTICAL predictor and an improvement of 3.66% compared to the conventional wide-band CSP approach. On the other hand, OPTICAL+ also achieved the highest average sensitivity and specificity of 70.10% and 69.10%, respectively. This shows that our proposed predictor is able to correctly classify more positive and negative samples, which in turn has resulted in the decrease in the error rate. Furthermore, OPTICAL+ achieved the highest κ value of 0.398, an improvement of 6.24% compared to that of the OPTICAL predictor. To add on, the proposed OPTICAL+ predictor achieved the lowest error rate for 21 out of the 52 subjects. This shows that apart from performing well in terms of the error rate, specificity, sensitivity and κ value, OPTICAL+ is also a robust and reliable predictor. The best performance by other methods in terms of the number of subjects that obtained the lowest error rate was by I-DFBCSP method (10 out of 52 subjects). This shows that the proposed predictor is more reliable as it performed well for most of the subjects on a larger dataset consisting of 52 subjects. Therefore, OPTICAL+ can be used to potentially develop improved HCI systems using EEG signals. Moreover, it can also be useful for other applications requiring EEG signal classification, such as various sleep stage classification and seizure detection.

## Supplemental Information

10.7717/peerj-cs.375/supp-1 Supplemental Information 1 Supplemental Material.

## Additional Information and Declarations

### Data Availability

The following information was supplied regarding data availability: Data is available at GigaScience: Cho H; Ahn M; Ahn S; Kwon M; Jun SC (2017): Supporting data for “EEG datasets for motor imagery brain computer interface” GigaScience Database. DOI 10.5524/100295 The database contains 52 Matlab files named s'#'.mat and it requires Matlab 2010a or a newer, preferably 64bit operating system. The source code is available at GitHub: https://github.com/ShiuKumar/OPTICAL_plus.
