# Supplementary material for: OPTICAL+: a frequency-based deep learning scheme for recognizing brain wave signals
Source: PeerJ Comput Sci. 2021 Feb 4;7:e375. doi: 10.7717/peerj-cs.375 (PMC7959638; doi:10.7717/peerj-cs.375)
Supplement: Supplemental Information 1 [file peerj-cs-07-375-s001.pdf]

Supplement 1 for OPTICAL+: A Frequency-Based Deep Learning Scheme for Recognizing Brain Wave Signals

Shiu Kumar[1], Ronesh Ashnil Sharma[1], Alok Sharma[2,3,4]

[1] Department of Electronic, Instrumentation & Control, Fiji National University, Suva, Fiji
[2] Department of Medical Science Mathematics, Medical Research Institute, Tokyo Medical and Dental University, Tokyo, Japan
[3] Laboratory for Medical Science Mathematics, RIKEN Center for Integrative Medical Sciences, Yokohama, Kanagawa, Japan
[4] Institute for Integrated and Intelligent Systems, Griffith University, Brisbane, QLD-4111, Australia

In this supplementary text, we present the 10x10-fold cross-validation results of the individual subjects for the different methods.

Table S1: Misclassification rate (%) of different methods evaluated using GigaDB dataset. The values after the ± sign are used to indicate the 95% confidence limits.

Subject	CSP	SBLFB	TFPO-CSP	I-DFBCSP	OPTICAL	OPTICAL+
1	**19.00 ± 2.56**	37.20 ± 2.94	22.00 ± 2.86	24.33 ± 2.73	20.00 ± 2.79	20.83 ± 2.35
2	48.20 ± 3.21	**44.10 ± 3.77**	48.50 ± 3.65	50.00 ± 3.61	47.67 ± 3.69	48.50 ± 2.77
3	8.70 ± 1.85	8.40 ± 2.16	6.17 ± 1.71	7.00 ± 1.78	6.00 ± 1.72	**4.67 ± 1.29**
4	26.50 ± 2.84	19.40 ± 2.64	19.33 ± 2.59	**10.17 ± 2.36**	22.00 ± 3.00	19.50 ± 2.80
5	**0.95 ± 0.49**	1.00 ± 0.60	1.17 ± 0.57	1.00 ± 0.61	1.00 ± 0.61	1.00 ± 0.61
6	19.50 ± 2.63	17.50 ± 2.43	18.33 ± 2.41	22.67 ± 2.56	17.67 ± 2.31	**14.17 ± 2.77**
7	48.42 ± 2.56	45.17 ± 2.67	49.58 ± 2.82	**44.44 ± 2.55**	47.22 ± 2.36	47.78 ± 3.21
8	47.40 ± 3.09	53.90 ± 3.57	47.50 ± 3.00	58.17 ± 3.22	**44.33 ± 3.47**	46.75 ± 3.18
9	44.00 ± 2.88	44.33 ± 2.96	48.19 ± 2.46	52.64 ± 2.10	43.60 ± 3.00	**39.58 ± 2.08**
10	31.10 ± 3.38	36.40 ± 3.00	23.83 ± 3.37	**20.17 ± 2.09**	27.00 ± 3.07	23.17 ± 2.70
11	50.00 ± 3.30	49.80 ± 3.03	49.17 ± 3.67	47.17 ± 3.34	42.67 ± 2.95	**41.50 ± 2.48**
12	33.50 ± 2.91	37.40 ± 3.06	34.17 ± 2.47	40.50 ± 2.96	32.83 ± 2.67	**32.67 ± 3.54**
13	19.40 ± 2.24	**11.50 ± 2.39**	11.17 ± 3.20	13.50 ± 2.22	15.50 ± 2.46	17.33 ± 2.13

14	4.60 ± 1.41	5.20 ± 1.59	4.33 ± 1.54	3.53 ± 1.18	3.50 ± 1.05	**3.17 ± 1.07**
15	41.30 ± 4.55	33.20 ± 3.51	42.17 ± 2.83	43.33 ± 4.04	35.33 ± 3.36	**27.17 ± 3.85**
16	55.00 ± 2.74	51.60 ± 2.99	53.33 ± 3.50	53.00 ± 3.91	52.50 ± 3.24	**46.83 ± 3.51**
17	49.20 ± 3.37	47.20 ± 3.22	49.00 ± 3.30	44.33 ± 3.73	50.33 ± 2.82	**42.83 ± 3.62**
18	49.40 ± 2.85	**41.10 ± 3.37**	50.17 ± 3.39	52.83 ± 3.07	46.67 ± 2.49	48.17 ± 2.78
19	45.00 ± 3.17	**35.40 ± 3.17**	45.00 ± 3.68	45.50 ± 3.78	42.83 ± 2.73	39.83 ± 3.07
20	28.90 ± 3.19	48.70 ± 3.20	32.17 ± 2.91	45.50 ± 3.82	**27.17 ± 3.02**	31.67 ± 3.24
21	41.10 ± 3.37	35.20 ± 3.23	41.00 ± 2.88	**26.17 ± 2.34**	34.00 ± 3.38	26.67 ± 3.51
22	46.80 ± 3.55	43.40 ± 3.49	41.17 ± 3.75	42.83 ± 3.27	41.33 ± 3.00	**39.50 ± 2.72**
23	21.70 ± 2.91	25.40 ± 2.10	15.83 ± 2.58	**10.50 ± 2.46**	15.83 ± 2.48	14.17 ± 1.84
24	45.50 ± 2.83	49.40 ± 2.74	43.67 ± 3.77	57.50 ± 3.17	39.33 ± 3.05	**33.33 ± 2.33**
25	50.50 ± 3.97	46.90 ± 3.42	48.50 ± 2.62	45.67 ± 2.92	47.00 ± 4.23	**43.50 ± 2.98**
26	4.40 ± 1.30	3.30 ± 1.11	4.33 ± 1.05	4.17 ± 1.36	**3.17 ± 1.00**	3.83 ± 1.28
27	57.10 ± 3.12	51.00 ± 3.13	**42.33 ± 3.10**	47.67 ± 2.90	55.33 ± 2.98	49.17 ± 2.69
28	20.80 ± 2.58	21.00 ± 2.41	20.33 ± 2.21	20.00 ± 3.03	19.17 ± 1.47	**18.67 ± 2.47**
29	56.20 ± 3.24	57.70 ± 3.12	52.83 ± 3.23	**50.67 ± 2.81**	57.00 ± 3.24	54.17 ± 3.06
30	42.80 ± 3.16	40.70 ± 3.56	**38.50 ± 3.16**	42.17 ± 3.47	44.50 ± 3.11	42.17 ± 4.12
31	42.60 ± 4.13	38.20 ± 3.67	42.50 ± 3.69	37.33 ± 3.27	37.67 ± 3.27	**32.17 ± 2.70**
32	50.10 ± 3.69	51.60 ± 3.66	50.50 ± 3.46	56.50 ± 3.35	49.43 ± 3.58	**49.00 ± 3.53**
33	48.00 ± 3.10	46.20 ± 3.03	51.33 ± 3.37	50.67 ± 2.73	**44.33 ± 3.64**	47.17 ± 3.47
34	44.50 ± 3.41	45.60 ± 2.99	44.67 ± 2.78	46.67 ± 3.16	**42.00 ± 2.23**	43.50 ± 3.57
35	20.20 ± 2.21	27.70 ± 3.47	21.33 ± 2.36	20.00 ± 2.11	**18.17 ± 2.78**	20.33 ± 2.47
36	34.10 ± 3.51	44.90 ± 3.53	36.00 ± 4.10	45.33 ± 4.37	**30.50 ± 3.04**	32.00 ± 2.58
37	22.50 ± 2.78	26.00 ± 2.62	**23.00 ± 3.24**	26.50 ± 2.42	23.02 ± 2.20	24.50 ± 3.35
38	52.30 ± 3.31	51.70 ± 2.79	46.67 ± 3.76	**46.00 ± 2.78**	51.50 ± 3.61	50.50 ± 2.83
39	29.40 ± 2.60	29.80 ± 3.52	33.67 ± 3.00	38.17 ± 3.17	27.00 ± 2.76	**26.83 ± 2.26**
40	49.70 ± 3.18	53.10 ± 3.05	**48.67 ± 3.55**	55.67 ± 2.56	48.83 ± 2.73	52.00 ± 3.56
41	16.40 ± 2.56	19.60 ± 3.10	16.00 ± 2.51	9.17 ± 1.80	14.67 ± 2.62	**13.33 ± 2.29**
42	53.90 ± 2.95	51.00 ± 3.27	53.00 ± 2.58	52.00 ± 3.31	51.67 ± 3.01	**51.17 ± 3.12**
43	5.90 ± 1.67	3.60 ± 1.35	3.83 ± 1.54	4.50 ± 1.32	4.17 ± 1.24	**2.50 ± 1.16**
44	11.10 ± 1.93	10.70 ± 1.93	**8.67 ± 1.92**	9.00 ± 1.97	10.83 ± 2.25	9.33 ± 1.91
45	46.40 ± 2.73	49.40 ± 3.76	**44.50 ± 2.01**	50.17 ± 3.07	47.50 ± 3.27	47.67 ± 3.29
46	25.25 ± 2.71	24.92 ± 2.73	24.44 ± 2.39	24.86 ± 2.98	25.42 ± 2.60	**21.25 ± 2.91**

47	24.60 ± 2.68	29.10 ± 2.62	30.33 ± 3.24	26.83 ± 2.46	25.83 ± 2.93	**21.67 ± 2.94**
48	36.70 ± 3.32	19.00 ± 2.52	26.67 ± 2.16	**11.82 ± 1.94**	21.83 ± 2.89	11.83 ± 2.36
49	14.20 ± 1.98	10.40 ± 1.47	12.17 ± 2.34	**8.67 ± 2.35**	12.50 ± 1.99	15.50 ± 2.69
50	0.00 ± 0.00	0.00 ± 0.00	0.00 ± 0.00	0.00 ± 0.00	0.00 ± 0.00	0.00 ± 0.00
51	47.30 ± 2.89	47.10 ± 2.89	47.17 ± 3.30	**46.33 ± 3.69**	46.83 ± 3.19	50.33 ± 3.71
52	39.70 ± 3.41	39.50 ± 2.92	**34.83 ± 3.05**	38.67 ± 2.95	38.17 ± 3.90	36.50 ± 3.70
Average	34.07 ± 2.80	33.88 ± 2.80	32.76 ± 2.78	33.31 ± 2.71	31.81 ± 2.78	**30.41 ± 2.70**
